# A network-based EEG source imaging framework for noninvasive localization of epileptogenic zones in MRI-negative focal drug-resistant epilepsy

**DOI:** 10.1186/s12883-026-04625-x

**Published:** 2026-01-15

**Authors:** Shicun Huang, Xiaowei Hu, Yiqing Wang, Wei Gao, Qi Fang

**Affiliations:** 1 Department of Neurology, The First Affiliated Hospital of Soochow University, 899 PingHai Road, Gusu District, Suzhou, Jiangsu 215000 China; 2 Department of Neurosurgery, The First Affiliated Hospital of Soochow University, 899 PingHai Road, Gusu District, Suzhou, Jiangsu 215000 China

**Keywords:** Drug-resistant epilepsy, MRI-negative epilepsy, EEG source imaging, Epileptogenic zone, Network analysis

## Abstract

**Objective:**

To develop and evaluate a dynamic brain network analysis framework integrating electroencephalography (EEG) source imaging (ESI) for non-invasive localization of the epileptogenic zone (EZ) in patients with magnetic resonance imaging (MRI)-negative focal drug-resistant epilepsy (DRE).

**Methods:**

We retrospectively analyzed 15 patients with MRI-negative focal DRE who underwent resective surgery. Preictal scalp EEG data were processed using ESI, followed by power spectral density and directed transfer function analyses to construct epileptic brain networks. Network metrics, including seizure index (SI), degree centrality (DC), out-degree centrality (DCout), and in-degree centrality (DCin), were calculated and evaluated for spatial concordance with postoperative resection zones and clinical outcomes.

**Results:**

Among the evaluated metrics, SI demonstrated the highest overall diagnostic performance, with a sensitivity of 60% and a specificity of 80%. DC and DCout showed high specificity (83.3%) but lower sensitivity, whereas DCin exhibited comparatively reduced sensitivity and accuracy.

**Conclusion:**

This non-invasive ESI-based network framework is capable of localizing epileptogenic regions in MRI-negative focal DRE. SI provided the most balanced performance across sensitivity and specificity, while DC and DCout contributed complementary information regarding epileptic network cores.

**Significance:**

The proposed framework offers a clinically feasible and non-invasive approach to support preoperative EZ localization, with potential value for presurgical evaluation in patients with MRI-negative epilepsy.

**Supplementary Information:**

The online version contains supplementary material available at 10.1186/s12883-026-04625-x.

## Introduction

Epilepsy is a heterogeneous neurological disorder characterized by complex pathophysiological mechanisms and diverse clinical manifestations. Despite the availability of more than 30 antiseizure medications (ASMs), approximately one-third of patients develop drug-resistant epilepsy (DRE), defined as persistent seizures despite adequate trials of two or more appropriately selected and tolerated ASMs [1]. For patients with focal DRE, surgical resection of the epileptogenic zone (EZ) remains the most effective therapeutic option, with seizure freedom achieved in approximately 60–80% of appropriately selected cases [2–4]. The EZ is classically defined as the cortical region responsible for seizure initiation and early propagation, and its complete resection is considered a key determinant of favorable postoperative outcomes [5–7]. However, increasing evidence suggests that surgical success depends not only on removal of the clinically apparent EZ but also on adequately addressing broader epileptogenic networks that may contribute to postoperative seizure recurrence if left untreated [5].

Magnetic resonance imaging (MRI)-negative epilepsy poses a particular and therapeutic diagnostic challenge, as conventional MRI fails to reveal overt structural abnormalities despite the presence of epileptogenic pathology [8]. In these subgroup, postoperative seizure freedom (Engel Class I) is achieved in only 40% to 50% of patients, compared to 70% to 80% in MRI-positive epilepsy [9]. Outcomes are particularly unfavorable in MRI-negative extratemporal, where surgical failure rates may exceed 50% [10]. These limitations largely reflect the restricted sensitivity of structural imaging and the inherent constraints of traditional scalp electroencephalography (EEG), both of which provide limited insight into the spatiotemporal dynamics of epileptogenic networks, thereby increasing uncertainty during presurgical evaluation.

Electroencephalographic source imaging (ESI) has emerged as a promising non-invasive approach for EZ localization in MRI-negative epilepsy. ESI reconstructs the cortical source activity underlying scalp EEG by solving both the forward problem, which models the conduction of electrical signals from the cortex to the scalp, and the inverse problem, which estimates the source distribution under biophysical constraints [11–14]. Modern distributed source models, such as standardized low-resolution brain electromagnetic tomography (sLORETA) [15], overcome the limitations of traditional single-dipole assumptions and allow whole-brain source estimation. Previous studies have demonstrated good concordance between ESI findings and intracranial EEG recordings, supporting its utility in presurgical localization, particularly in MRI-negative cases [15–20].

Concurrently, advances in computational neuroscience have increasingly conceptualized epilepsy as a network disorder, in which seizures arise from abnormal dynamic interactions among distributed brain regions rather than from a single focal source [21, 22]. This paradigm shift has led to the integration of functional connectivity (FC) analysis with ESI, enabling characterization of network-level properties such as node centrality, hubness, and directed information flow. Such approaches facilitate identification of critical network nodes and seizure propagation pathways that may not be apparent from focal source localization alone [23–26]. Notably, FC analyses based on source-reconstructed EEG have been reported to improve localization performance in MRI-negative epilepsy, with localization accuracies exceeding 75% in selected cohorts [27–30].

As MRI-negative epilepsy represents an increasingly large proportion of candidates referred for epilepsy surgery [31], there is a growing need for individualized, clinically feasible, and network-informed localization strategies. In this study, we propose a multidimensional ESI-based framework that integrates power spectral density (PSD), directed transfer function (DTF), and graph-theoretical degree centrality metrics to characterize epileptogenic networks from scalp EEG. In addition, we incorporate the seizure index (SI) as a dynamics measure to capture temporal variations in seizure propensity across network nodes. By combining source-level localization with network and dynamical analyses, this framework aims to support non-invasive EZ localization and to provide complementary information for presurgical evaluation in patients with MRI-negative focal DRE.

## Methods

### Patients

This single-center retrospective study included 15 patients with MRI-negative focal DRE who underwent epilepsy surgery at the First Affiliated Hospital of Soochow University between June 2019 and February 2023. The study protocol was approved by the local ethics committee (approval No. 2024377). Written informed consent was obtained from all participants prior to inclusion. All procedures were conducted in accordance with the Declaration of Helsinki, and all clinical and EEG data were anonymized before analysis.

#### Inclusion and exclusion criteria

Patients were eligible for inclusion if they met all of the following criteria: (1) diagnosis of focal DRE according to the International League Against Epilepsy (ILAE) 2010 criteria [32, 33]; (2) completion of a comprehensive presurgical evaluation including video-EEG (VEEG), high-resolution 3.0-T MRI, and ^18^F-fluorodeoxyglucose positron emission tomography–computed tomography (^18^F-FDG PET-CT), with no definite epileptogenic lesion identified on MRI; (3) inconclusive EZ localization following noninvasive (Phase I) evaluation and multidisciplinary team (MDT) discussion; (4) subsequent implantation of stereo-electroencephaloghy (SEEG) electrodes and resective surgery, with complete clinical and follow-up data available; (5) provision of written informed consent. Exclusion criteria were as follows: (1) severe cerebral atrophy affecting cortical surface reconstruction; (2) generalized epilepsy; (3) absence of epileptiform discharges during SEEG monitoring; (4) contraindications to epilepsy surgery; (5) incomplete clinical, EEG, imaging, or follow-up data.

#### Presurgical evaluation and postoperative outcome assessment

All patients underwent a standardized presurgical evaluation protocol comprising noninvasive (Phases 1a/1b) and invasive (Phase 2) assessments (Supplementary Fig. 1). Phase 1a evaluation included detailed clinical history, long-term VEEG monitoring, 3.0-T MRI, and neuropsychological tests. For MRI-negative cases, additional Phase 1b investigations were performed, including MRI post-processing and ^18^F-FDG PET-CT. Patients with discordant or inconclusive noninvasive findings proceeded to Phase II evaluation with SEEG implantation to establish electroclinical correlation and to confirm the EZ.

All patients subsequently underwent individualized resective surgery. Postoperative follow-up was conducted for at least 12 months. Seizure outcomes were classified according to the Engel outcome scale, with Engel class I considered a favorable outcome and classes II–IV considered unfavorable. Follow-up assessments included clinical evaluations, antiseizure medication management, routine EEG, and postoperative MRI.

This study retrospectively analyzed presurgical scalp EEG data using ESI, and the resulting EZ localization was compared with postoperative resection sites and clinical outcomes.

### EEG recordings and head modeling

#### EEG acquisition and preprocessing

Seizure activity is commonly classified into interictal, preictal, ictal, and postictal phases based on clinical manifestations and corresponding EEG patterns [34] (Supplementary Fig. 2). Only patients who exhibited at least one complete seizure during EEG monitoring were included in the analysis. EEG data were recorded using a Nihon Kohden EEG-1200C system with 21 scalp electrodes positioned according to the international 10–20 system. Signals were bandpass filtered between 0.5 and 80 Hz, and 50 Hz line noise was removed. Independent component analysis (ICA) was applied to remove ocular and electrocardiographic artifacts. For each seizure, EEG dynamics were analyzed with a specific focus on the 10-min interval preceding seizure onset, hereafter referred to as the “long preictal period.” This extended window was selected to capture slowly evolving pre-seizure network dynamics beyond the short preictal intervals typically used in routine clinical EEG interpretation. Only seizures with complete ictal recordings were retained for analysis (Supplementary Fig. 3).

#### Head model construction

High-resolution T1-weighted MRI scans were acquired using a 3.0-T GE Signa Premier scanner with a magnetization-prepared rapid acquisition gradient-echo (MPRAGE) sequence. Individualized realistic head models were constructed using the boundary element method (BEM) based on tissue segmentation of the scalp, skull, cerebrospinal fluid and brain. Tissue segmentation was performed using CAT12 (Computational Anatomy Toolbox 12) within SPM (Statistical Parametric Mapping). MRI data were converted to Neuroimaging Informatics Technology Initiative (NIfTI) format, spatially normalized to Montreal Neurological Institute (MNI) space, and smoothed to enhance spatial fidelity (Supplementary Fig. 4).

### EEG source imaging and network analysis

An overview of the ESI-based localization framework is shown in Supplementary Fig. 5. EZ localization was performed by integrating source-level power analysis with directed network metrics derived from reconstructed cortical activity. First, sLORETA was applied to preprocessed scalp EEG data to solve the inverse problem and estimate the spatiotemporal distribution of cortical source current density. Source-level time series were subsequently extracted across multiple frequency bands for further network analysis.

#### EEG source imaging

Source localization was performed using sLORETA implemented in Brainstorm [35]. Realistic BEM [17] head models were generated, and electrode positions were co-registered to individual cortical surfaces. Cortical reconstruction was performed using FreeSurfer [36], yielding 15,002 vertices per hemisphere. The cortex was parcellated into 68 regions based on the Desikan–Killiany atlas [37] (Supplementary Table 1).

#### Power spectral density

PSD was computed from source-level time series using a fast Fourier transform (FFT) with a 2-s sliding window. This window length was selected to balance spectral reliability and temporal resolution, allowing detection of transient spectral changes while preserving sensitivity to slow network dynamics across the long preictal period. PSD values were log-transformed(dB/Hz) and calculated for six conventional frequency bands (δ, θ, α, β, low-γ, high-γ). Regional PSD values were obtained by averaging across vertices within each cortical region.

#### Directed transfer function and graph-theoretical analysis

Directed functional networks were constructed in source space using the DTF to quantify directional information flow between cortical regions. Graph-theoretical metrics were subsequently calculated to characterize network topology. DC was defined as the total number of connections incident on a node, or the sum of edge weights in weighted networks, reflecting its global network involvement [38]. In directed networks, DCout quantified the extent to which a node exerted influence over other regions and was interpreted as a potential driver of epileptic activity, whereas DCin reflects the extent to which a node receives inputs and was associated with seizure propagation [39]. DC, DCin and DCout were calculated across a range of sparsity thresholds (Supplementary Fig. 6). The area under the curve (AUC) across thresholds was used to identify network hubs, with normalized values ≥ 0.95 considered significant.

#### Seizure index

The SI was used to quantify regional seizure propensity based on a bistable dynamical system model derived from DTF-based networks [40, 41]. Hilbert-transformed signals were used to obtain instantaneous amplitude and phase information. SI was defined as the inverse of the mean escape time from a stable state to a seizure state, with higher SI values indicating increased local instability and a greater propensity for seizure generation. Regions with normalized SI values ≥ 0.95 were considered highly epileptogenic.

### Reference standard

The reference standard for EZ localization was defined as the surgically resected brain region associated with postoperative seizure freedom (Engel Class I) after a minimum follow-up of 12 months. Localization was considered accurate when the predicted EZ spatially overlapped with the resection area and was associated with an Engel class I outcomes [42].

Cases were classified as true positives (TP) when the predicted EZ overlapped the resection zone and resulted in Engel class I outcomes; false positives (FP) when localization overlapped the resection zone but did not result in Engel class I outcomes; false negatives (FN) when localization did not overlap the resection zone despite Engel class I outcomes; and true negatives (TN) when localization did not overlap the resection zone and outcomes were unfavorable.

### Outcome measures

Diagnostic performance was evaluated using sensitivity, specificity, accuracy, positive likelihood ratio (PLR), negative likelihood ratio (NLR),and diagnostic odds ratio (DOR), calculated as follows:

$$\mathrm{Sensitivity}=\frac{TP}{TP+FN}$$

$$\mathrm{Specificity}=\frac{TN}{TN+FP}$$

$$\mathrm{Accuracy}=\frac{TP+TN}{TP+FP+TN+FN}$$

$$\text{Diagnostic Odds Ratio, DOR}=\frac{TP\times TN}{FP\times FN}$$

$$\text{Positive Likelihood Ratio},\text{ PLR}=\frac{\mathrm{Sensitivity}}{1-\mathrm{Specificity}}$$

$$\text{Negative Likelihood Ratio, NLR}=\frac{1-\mathrm{Sensitivity}}{\mathrm{Specificity}}$$

### Statistical

For all diagnostic accuracy metrics, 95% confidence intervals (CIs) were calculated. Differences in localization performance among DC, DCin, DCout, and SI were assessed using the chi-square test or Fisher’s exact test, as appropriate, based on TP, FP, TN, and FN counts. All statistical analyses were performed using R software (version 4.3.2; R Foundation for Statistical Computing, Vienna, Austria).

## Results

### Clinical characteristics

This retrospective cohort comprised 15 patients with MRI-negative focal DRE who underwent SEEG-guided resective surgery and completed at least 12 months of postoperative follow-up. All patients received individualized SEEG implantation and both ictal and interictal recordings. Surgery strategies were determined based on SEEG findings, and postoperative CT and pathology examinations were used to confirm the extent of resection and assess procedure-related complications (Supplementary Fig. 7).

Demographic and clinical characteristics are summarized in Supplementary Table 2. The cohort included 3 males (20%) and 12 females (80%), with a mean age of 28.53 ± 7.27 years and a mean epilepsy duration of 13.27 ± 7.85 years. According to the ILAE 2017 classification, 9 patients were diagnosed with temporal lobe epilepsy (TLE) and 6 with extratemporal epilepsy. Postoperative seizure outcomes were classified as Engel class I in 10 patients (66.7%), Class II in 2 patients (13.3%), and Class IV in 3 patients (20.0%). No significant difference in Engel class I outcome was observed between TLE and extratemporal epilepsy (*p* > 0.05).

### Power spectral density analysis

Analysis of preictal scalp EEG revealed a consistent increase in delta-band (1–4 Hz) power across patients during the long preictal period (Supplementary Fig. 8). Based on this observation, subsequent source-space network analyses focused primarily on the delta frequency band.

### Directed transfer function-based centrality analysis

**Fig. 1:**
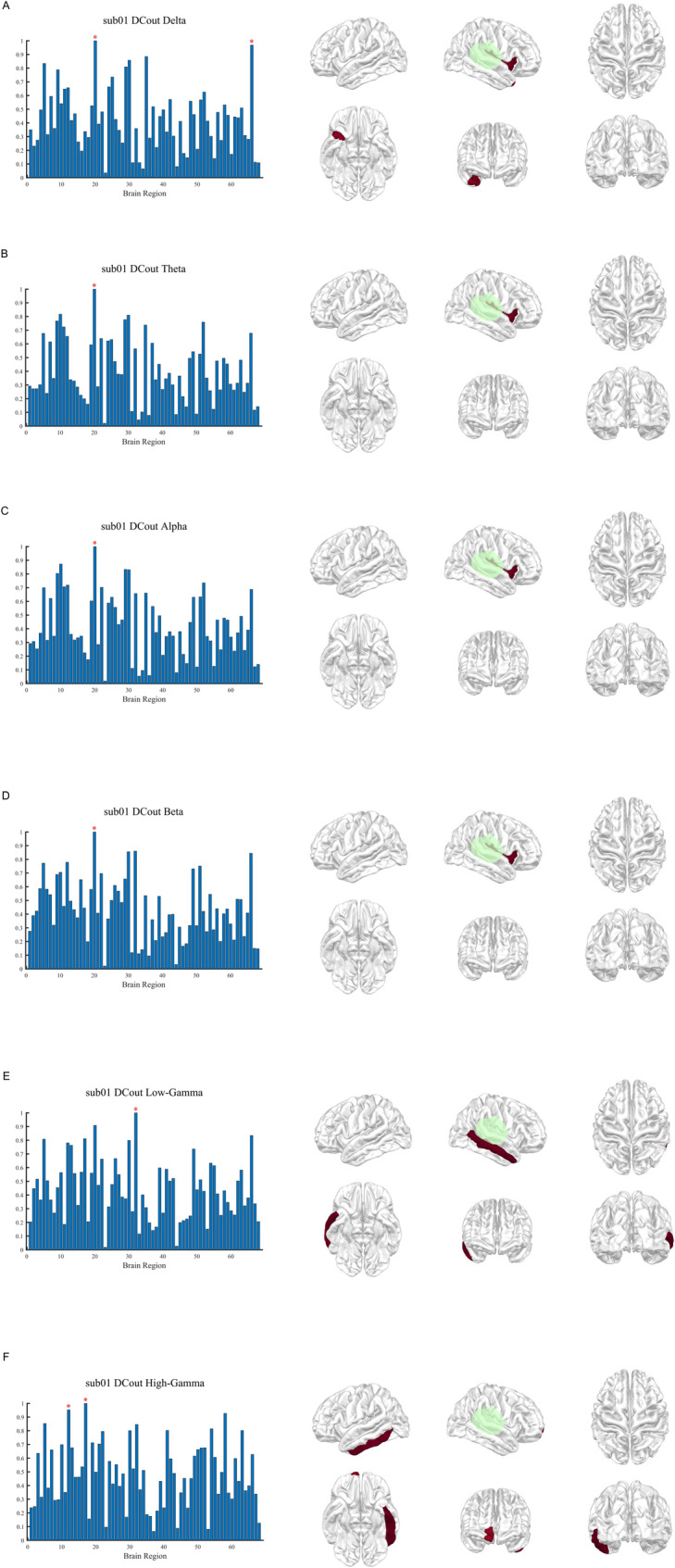
DCout distribution across frequency bands in patient sub01. Patient sub01 is presented as an illustrative example to demonstrate DCout distributions across six frequency bands (δ, θ, α, β, low-γ, and high-γ). For each frequency band, the left panel displays a bar plot of normalized DCout values across 68 cortical regions (x-axis: region index; y-axis: normalized DCout value). Regions with normalized DCout values ≥ 0.95 are marked with red asterisks. The right panel shows the corresponding cortical surface maps from six standard views (left, right, dorsal, ventral, rostral, caudal), with warmer colors indicating higher DCout intensity. Surgically resected regions are outlined in green

**Fig. 2:**
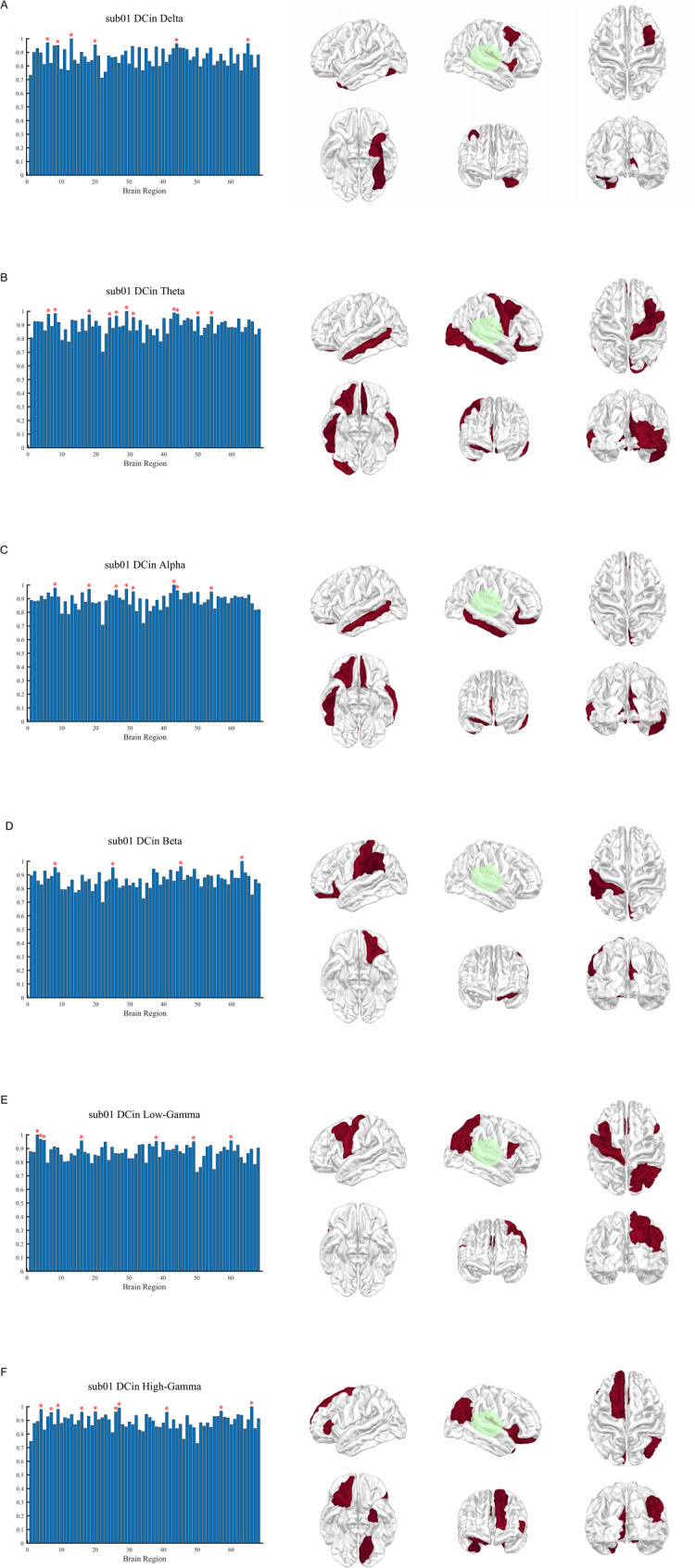
DCin distribution across frequency bands in patient sub01. Normalized DCin distribution for patient sub01 across six frequency bands (δ to γ). For each band, the left panel presents bar plots of normalized DCin values across 68 cortical regions, with regions ≥ 0.95 indicated by red asterisks. The right panel illustrates spatial DCin distributions from multiple cortical views, with warmer colors representing higher DCin values. Green outlines indicate the surgically resected regions

**Fig. 3:**
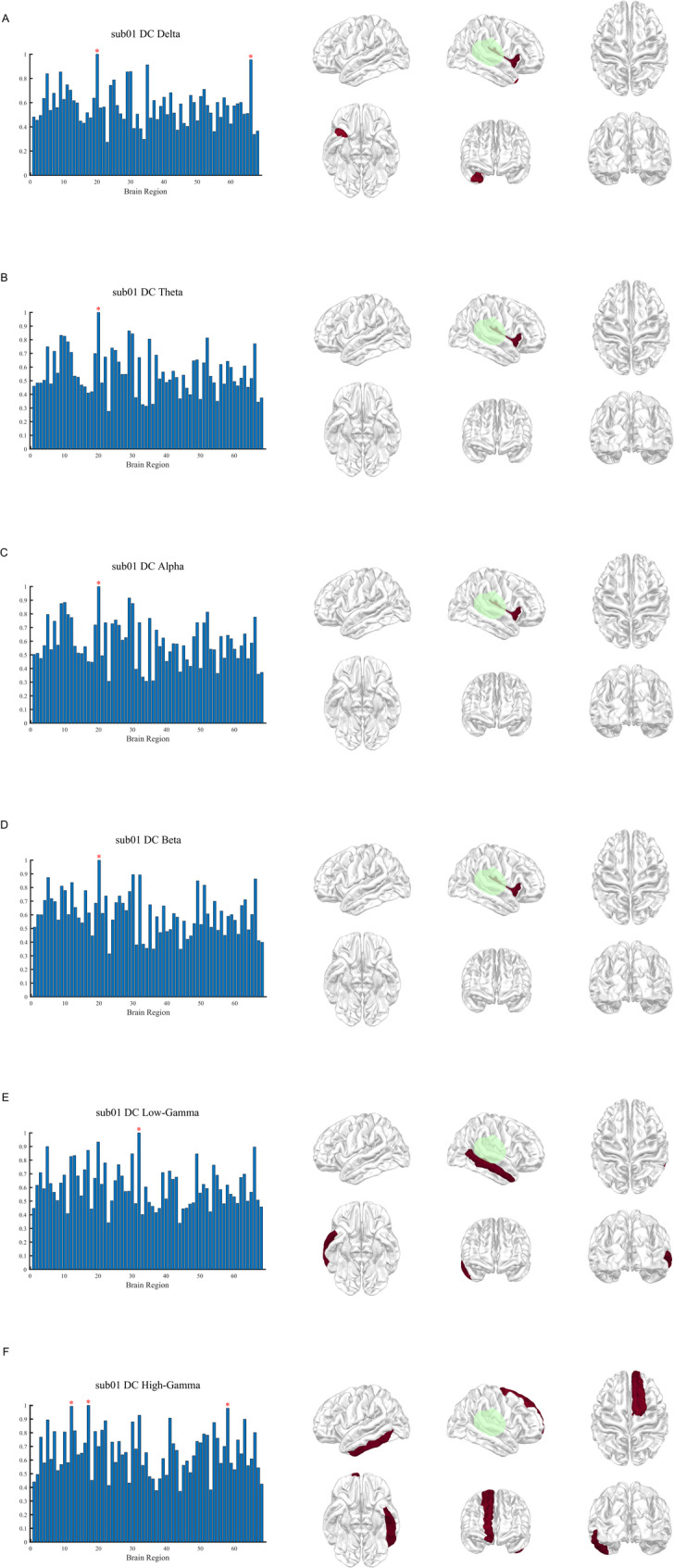
DC distribution across frequency bands in patient sub01. DC distribution across six frequency bands (δ to γ) in patient sub01. For each frequency band, the left panel shows bar plots of normalized DC values across cortical regions, highlighting regions ≥ 0.95 with red asterisks. The right panel presents cortical surface visualizations from six standard views, with warmer colors indicating higher DC values. Surgically resected regions are highlighted in green

**Fig. 4:**
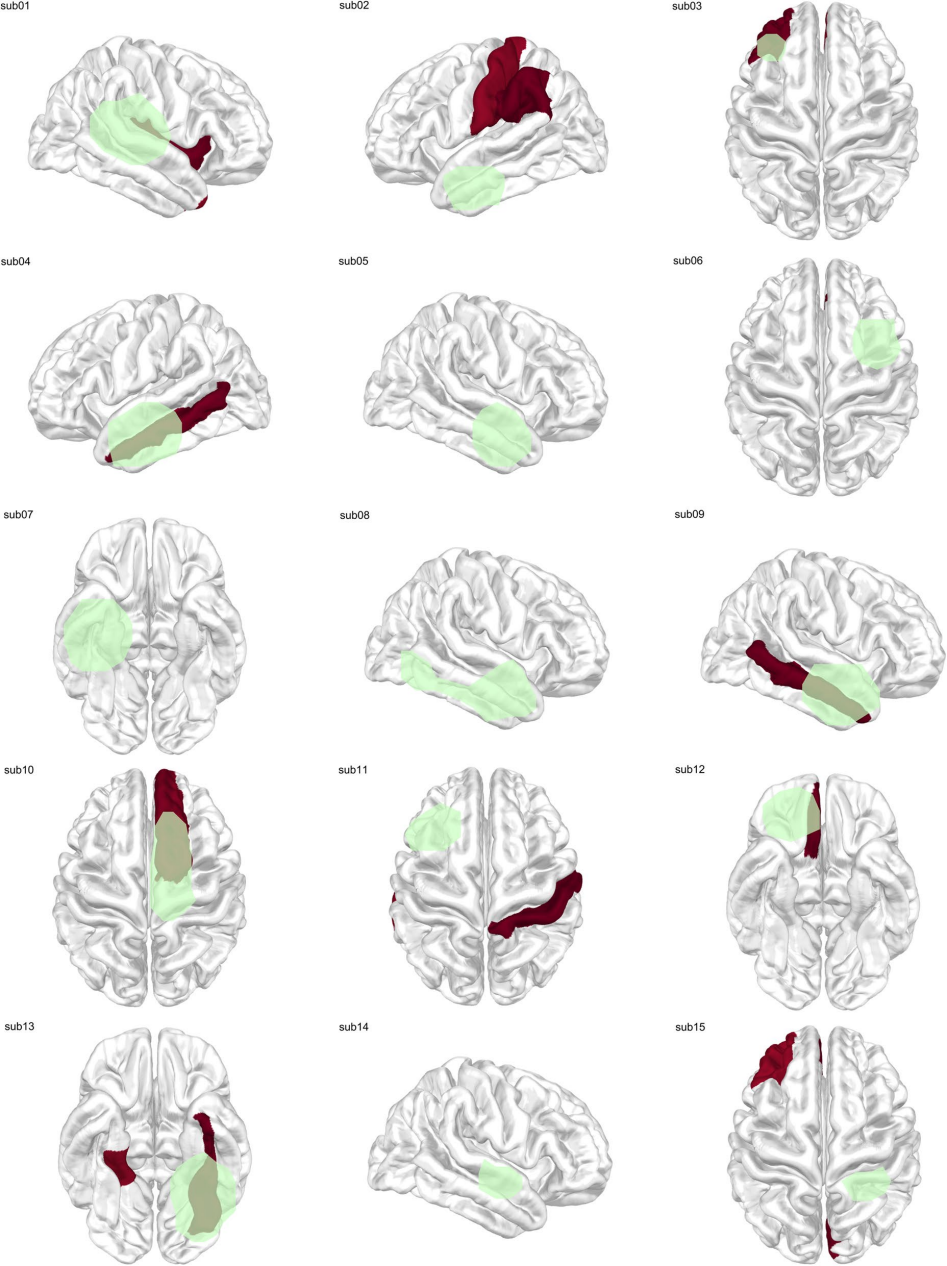
Group-level DCout distribution in the delta band. Group-level distribution of delta-band DCout values across 15 patients. Cortical regions with normalized DCout values ≥ 0.95 are highlighted in red, with darker shading indicating higher centrality values. Green outlines denote brain regions that were surgically resected in each patient

DTF analysis was applied to source-level EEG data across six frequency bands. Degree-based centrality metrics, including DC, DCout, and DCin were calculated, normalized, and visualized for regions with values within the top 5%. In an illustrative example (patient sub01), DCout showed a strong spatial correspondence with the surgically resected regions in low-frequency bands (delta to beta), whereas this concordance was less apparent in gamma-band networks (Fig. 1). In contrast, DCin highlighted several regions outside the resection area, suggesting involvement in seizure propagation rather than seizure onset (Fig. 2). DC largely overlapped with DCout patterns but additionally revealed focal activity in higher frequency bands not consistently captured by DCout (Fig. 3). At the group level, delta-band DCout demonstrated concordance with the resection zones in 7 of 15 patients, including 4 cases of TLE and 3 cases of frontal lobe epilepsy, indicating consistent localization patterns across both temporal and extratemporal epilepsies (Fig. 4).

### Seizure index analysis

**Fig. 5:**
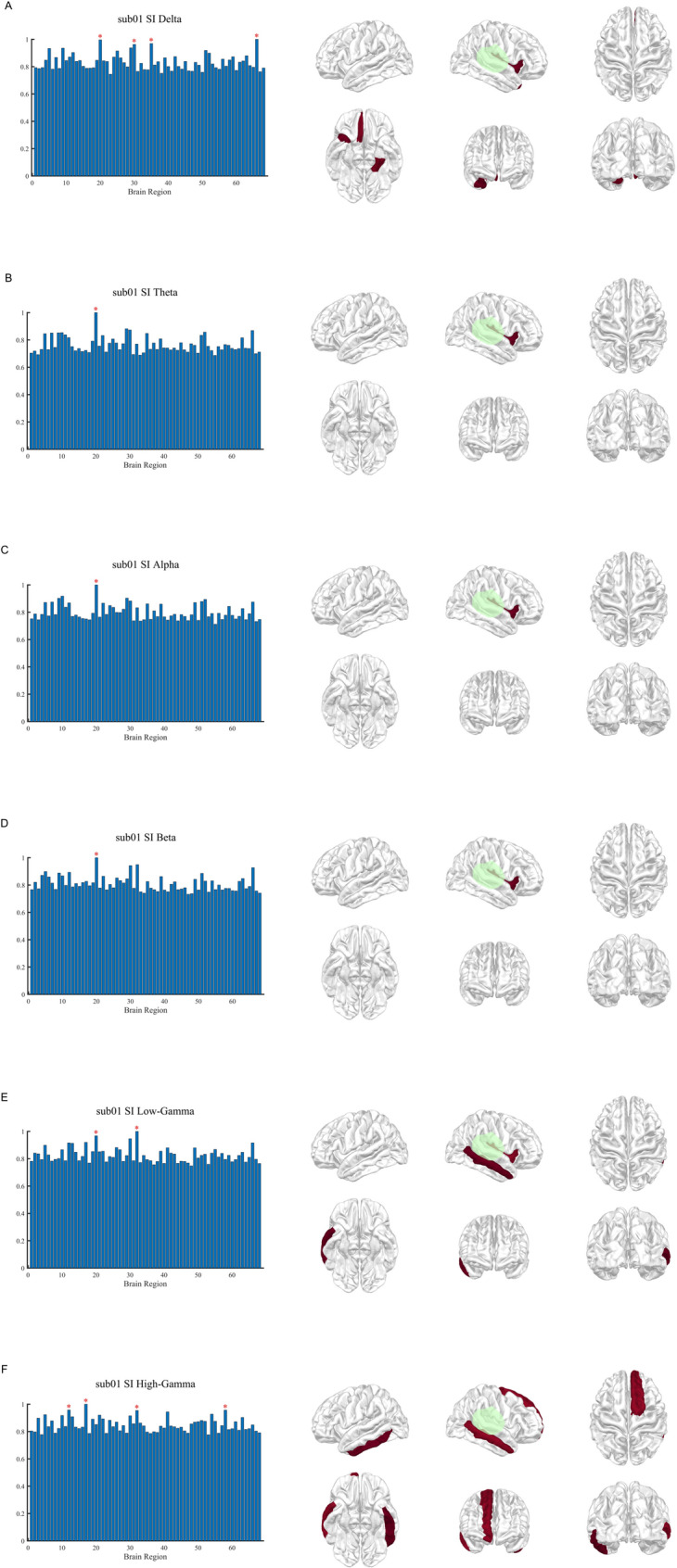
Seizure index distribution across frequency bands in patient sub01. Patient sub01 is shown as an illustrative example of SI distributions across six frequency bands (δ, θ, α, β, low-γ, and high-γ). For each frequency band, the left panel presents bar plots of normalized SI values across 68 cortical regions, with candidate epileptogenic regions (SI ≥ 0.95) marked by red asterisks. The right panel displays cortical surface maps from six standard views, with warmer colors indicating higher SI values. Surgically resected regions are highlighted in green

**Fig. 6:**
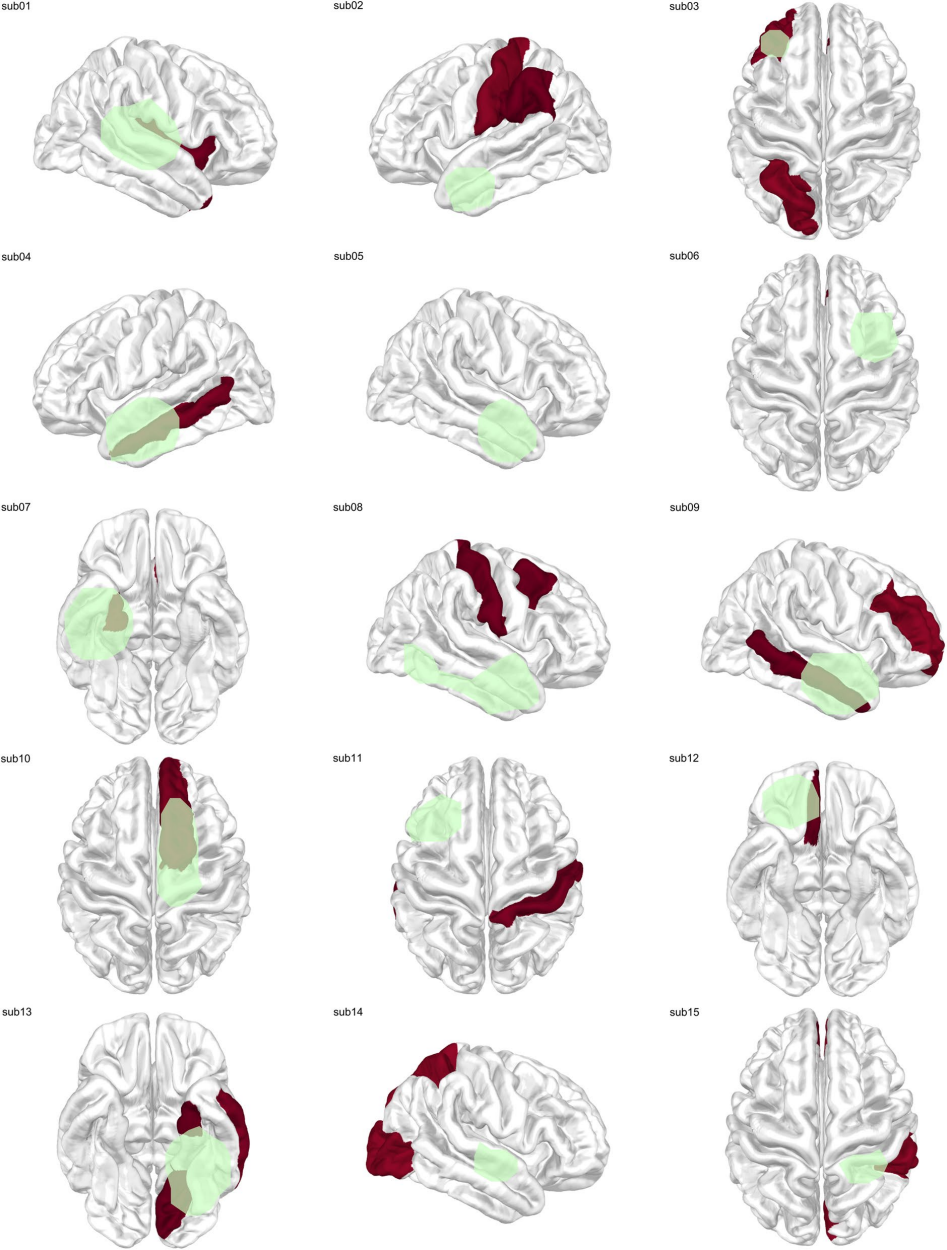
Group-level SI distribution in the delta band. Group-level cortical distribution of delta-band SI values across 15 patients. Regions with normalized SI values ≥ 0.95 are shown in red, with darker shading indicating higher seizure propensity. Green outlines indicate surgically resected regions

SI values were computed across six frequency bands using Hilbert-transformed source signals. Brain regions with normalized SI values ≥ 0.95 were identified as candidate epileptogenic zones. In patient sub01, elevated SI values in low-frequency bands spatially overlapped with the surgically resected regions (Fig. 5). Across the entire cohort, delta-band SI identified regions concordant with resection zones in 8 of 15 patients, including 5 cases of TLE and 3 cases of frontal lobe epilepsy (Fig. 6). Notably, in one patient, delta-band SI detected concordant regions that were not identified by DCout analysis.

### Comparison of diagnostic performance

The diagnostic performance of four network metrics, including SI, DCout, DC, and DCin, was quantitatively compared using sensitivity, specificity, accuracy, PLR, NLR, and DOR (Supplementary Table 3). SI demonstrated the highest sensitivity (60%, 95% CI: 31.3–83.2%) and the lowest NLR (0.5, 95% CI: 0.12–2.00), indicating a relatively stronger ability to detect epileptogenic zones and reduce false-negative localization. Its specificity was moderate (80%, 95% CI: 37.6–96.4%).

Both DCout and DC showed the highest specificity (83.3%, 95% CI: 43.6–97.0%), accuracy (66.7%, 95% CI: 41.7–84.8%), and DOR (6.25, 95% CI: 2.18–17.96). Their PLR values exceeded 3, suggesting a higher probability of correct EZ localization when test results were positive. In contrast, DCin exhibited lower sensitivity (40%, 95% CI: 16.8–68.7%), higher NLR (0.75, 95% CI: 0.21–2.66), and the lowest DOR (2.67, 95% CI: 1.04–6.82), indicating limited standalone value in this cohort.

All metrics were associated with relatively wide confidence intervals, reflecting the limited sample size. Nevertheless, the observed results indicate complementary diagnostic profiles, with SI favoring sensitivity and DC-based metrics emphasizing specificity.

## Discussion

Approximately 30% of patients with epilepsy develop DRE [43], for whom surgical resection remains one of the most effective treatment options [44–46]. Accurate localization of the EZ is therefore essential for achieving favorable postoperative outcomes [10, 45]. Despite advances in multimodal presurgical evaluation, precise EZ identification remains challenging [5], particularly in MRI-negative epilepsy, where structural imaging fails to reveal overt lesions and additional functional imaging or invasive monitoring is often required [6, 47, 48]. As a result, only a subset of patients complete the full surgical evaluation pathway, and among those who undergo surgery, approximately two-thirds achieve sustained seizure control [49, 50]. To address these challenges, increasing attention has been directed toward advanced EEG signal analysis techniques, including ESI. Compared with other functional imaging modalities, ESI and magnetoencephalography (MEG) directly capture epileptiform electrical activity with high temporal resolution. In the present study, we applied ESI to conventional scalp EEG recordings from patients with MRI-negative focal DRE and integrated SI and degree-based network metrics (DCout and DC) within a unified analytical framework. Our results demonstrate the feasibility of this approach and suggest that these quantitative indices can provide complementary information for noninvasive EZ localization.

With the evolution of computational neuroscience, ESI has evolved from a primarily visual localization tool to a spatiotemporal analytical framework incorporating individualized head models and advanced inverse solutions [51–58]. Multiple studies have reported good spatial concordance between ESI findings, invasive EEG recordings, and surgical resection zones, particularly in MRI-negative epilepsy [54, 56, 59–72]. In some cohorts, ESI-based localization has been associated with favorable postoperative seizure outcomes [73]. These observations support the growing role of ESI as a valuable component of presurgical evaluation, especially when structural imaging is inconclusive.

In the present cohort, spatial concordance between ESI-derived metrics and the resection zone was observed in 60% of cases for SI and in 55.6% of cases for DCout and DC. The slightly lower concordance compared with some previous reports may be attributable to several factors. tFirst, we relied on conventional 21-channel scalp EEG rather than high-density EEG (HD-EEG), which offers improved spatial sampling and may enhance source localization accuracy. Second, differences in postoperative seizure outcome distributions across studies may influence apparent concordance rates, as seizure freedom itself is closely linked to the completeness of EZ resection. These considerations highlight the importance of interpreting ESI performance within the context of recording methodology and clinical outcome profiles.

A notable finding of this study was the consistent localization performance observed in the delta (δ) frequency band. Delta-band activity is widely recognized as a marker of pathological cortical dysfunction in epilepsy, reflecting impaired inhibitory control, cortical deafferentation, and large-scale network instability [74, 75]. Prior studies have shown that focal δ slowing often colocalizes with seizure onset regions and corresponds to areas of metabolic dysfunction on ^18^F-FDG PET, even in MRI-negative patients [75, 76]. From a network perspective, low-frequency oscillations capture slow, spatially extended synchronization patterns that may better reflect the core epileptogenic network than transient or spatially restricted high-frequency activity.

In addition, low-frequency signals provide favorable conditions for EEG source reconstruction due to their higher signal-to-noise ratio and reduced susceptibility to muscle artifacts and volume conduction effects. Source imaging methods such as sLORETA have been shown to yield more stable and reliable estimates in lower frequency ranges, whereas higher-frequency activity is more sensitive to noise and modeling uncertainties [15, 19]. These methodological characteristics likely contribute to the relatively robust performance of δ-band–based metrics observed in this study.

Seizure generation is increasingly understood as a dynamic process involving gradual transitions between interictal, preictal, ictal, and postictal states [77]. During the preictal period, progressive changes in spectral content, neuronal synchronization, and functional and effective connectivity can be detected minutes before seizure onset [78–83]. These findings suggest that the preictal brain occupies a metastable state characterized by evolving network instability. Accordingly, the long preictal window analyzed in this study was designed to capture slow network-level transitions, while the shorter sliding-window spectral analysis allowed for fine-grained temporal sampling. This multiscale strategy enables the characterization of gradual seizure-related dynamics without assuming abrupt preictal changes.

Although preictal EEG has been extensively investigated in seizure prediction research [73, 84, 85], it has been less frequently explored in the context of EZ localization. Most prior ESI studies have focused on interictal or ictal recordings, with systematic reviews suggesting that ictal ESI provides the highest diagnostic performance [42]. By extending ESI-based network analysis to the preictal period, the present study introduces a complementary noninvasive approach that may enrich existing presurgical localization paradigms, particularly in MRI-negative cases where multiple sources of evidence are required.

From a network perspective, degree centrality reflects a node’s overall involvement in information transfer. DCout has been associated with seizure onset regions, whereas DCin is more closely related to propagation pathways. The SI, derived from dynamical modeling, characterizes regional instability and seizure propensity. In this study, δ-band SI demonstrated slightly higher sensitivity for spatial concordance with the resection zone, whereas DCout and DC provided higher specificity and insight into network driving patterns. These findings suggest that SI and degree-based metrics capture complementary aspects of epileptogenic networks.

From a clinical standpoint, false-negative localizations were more frequently observed in cases involving deep-seated foci, distributed epileptogenic networks, or rapid seizure propagation—well-recognized limitations of scalp EEG–based methods. While SI showed favorable localization characteristics, its potential utility in identifying residual epileptogenic tissue or predicting postoperative seizure recurrence warrants further investigation. Importantly, the proposed framework is intended as a quantitative, semi-automated decision-support tool that complements expert clinical judgment rather than replacing established presurgical evaluation strategies.

Building on the observed complementarity between SI and degree-based metrics, future studies may explore composite or multivariate models that integrate multiple network features to improve localization robustness. In the present study, we deliberately focused on the independent diagnostic contribution of each metric and avoided multivariate modeling due to the limited sample size and the associated risk of overfitting. Larger prospective cohorts will be required to evaluate integrated models and to validate their clinical utility.

In the broader context of epilepsy as a network disorder, the integration of ESI with directed and topological network metrics represents a promising noninvasive strategy for presurgical mapping and SEEG planning in MRI-negative DRE. Nevertheless, ESI remains constrained by volume conduction effects and reduced sensitivity for deep or bilateral epileptogenic regions. Accordingly, ESI-based network analysis is best positioned as an initial localization tool that should be interpreted alongside other modalities, such as PET, fMRI, MEG, and invasive recordings, to support individualized surgical decision-making.

## Limitation

Several limitations of this study should be acknowledged. First, the retrospective design may have introduced inherent biases related to data completeness, quality control, and outcome assessment. Prospective studies are therefore required to further validate the robustness and reproducibility of the proposed framework. Second, the relatively small sample size limited statistical power and generalizability. In particular, the limited number of patients with temporal and extratemporal epilepsy precluded formal subgroup comparisons; accordingly, any observed differences between epilepsy subtypes should be interpreted as exploratory.

Third, the long preictal interval adopted in this study differs from the conventionally defined clinical preictal window. While this approach was intentionally designed to capture gradual network reconfiguration preceding seizure onset, its optimal temporal definition and direct clinical applicability require further validation. Fourth, multivariate integration of complementary network metrics was not performed in order to minimize the risk of overfitting in a small cohort. Future studies with adequately powered samples should explore composite or multivariate models to assess whether integrated approaches can further improve localization accuracy.

Fifth, as an inverse problem–based technique, ESI is inherently constrained by model assumptions, head model accuracy, and algorithmic parameters, and the resulting source estimates represent probabilistic reconstructions rather than direct measurements. Sixth, the use of low-density scalp EEG may have limited spatial resolution; future investigations incorporating high-density EEG are warranted to assess potential gains in localization performance.

Finally, direct validation against SEEG–defined EZs was not performed in this study. Nevertheless, previous work has demonstrated substantial spatial concordance between ESI and SEEG findings, particularly in MRI-negative epilepsy and focal cortical malformations [73, 86–88]. Future studies integrating simultaneous or comparative ESI–SEEG analyses would further strengthen the clinical validation of this approach.

Despite these limitations, the present study establishes a structured and quantitative framework for integrating directed network analysis with ESI in MRI-negative drug-resistant epilepsy and provides a foundation for future investigations aimed at improving noninvasive presurgical localization strategies.

## Conclusion

In this study, we propose a noninvasive framework for epileptogenic zone localization that integrates dynamic EEG source imaging with time-varying network analysis in patients with MRI-negative focal drug-resistant epilepsy. By combining seizure index and degree-based network metrics, this approach captures complementary aspects of epileptogenic network organization, including intrinsic seizure propensity and directional network driving properties.

The results suggest that seizure index may enhance sensitivity for identifying candidate epileptogenic regions, while degree-based metrics provide higher specificity and insight into network structure. Together, these findings support the potential clinical value of ESI-based network analysis as a quantitative decision-support tool for presurgical evaluation in MRI-negative epilepsy.

Future work should focus on prospective validation in larger, multicenter cohorts, integration of complementary network features using appropriately powered multivariate models, and incorporation of structural connectivity information to further refine network representations. Such efforts may help advance noninvasive, quantitative strategies for presurgical assessment and facilitate their translation into routine clinical practice.

## Supplementary Information

Supplementary Material 1: Supplementary Table 1. Desikan-Killiany Atlas. Supplementary Table 2. Baseline Characteristics of Patients. Supplementary Table 3. Diagnostic Outcome Indicators. Supplementary Fig. 1. Preoperative assessment flowchart. Note: Stage 1a: All patients initially undergo standard non-invasive evaluations. If findings are conclusive, surgical intervention may proceed directly. Stage 1b: For inconclusive or discordant results, additional advanced non-invasive investigations may be performed to refine EZ localization or assess surgical risk. The selection of modalities depends on clinical context and institutional resources. Stage 2: If stage 1b results remain non-localizing or suggest that the EZ is adjacent to eloquent cortex, SEEG combined with cortical stimulation is employed to delineate both the EZ and functional areas. This study involved a retrospective collection of patients’ scalp EEG data and the localization of EZ using ESI, which were subsequently evaluated for concordance with postoperative resection sites and clinical prognosis. Supplementary Fig. 2. Stages of seizure. Note: The horizontal axis represents time, and the vertical axis denotes EEG channel labels. The timeline includes the interictal, preictal, ictal, and postictal phases. In this study, a 10-min segment of EEG data preceding seizure onset—highlighted by red brackets and indicated with a red star—was selected for further analysis. Supplementary Fig. 3. EEG preprocessing. Note: (a) A 10-min EEG segment preceding seizure onset was selected; (b) electrode channel positions were accurately aligned according to the 10–20 system; (c) a band-pass filter (0.5–80 Hz) and a notch filter (48–52 Hz) were applied to remove baseline drift and power line interference, respectively; (d) independent component analysis (ICA) was conducted to isolate noise components; (e) artifacts and identified interference signals were removed; and (f) the cleaned EEG data were segmented into 2-s epochs, ensuring minimal residual noise and optimal signal quality for subsequent analysis. Supplementary Fig. 4. Head models. Note: (a) Acquisition of T1-weighted magnetic resonance imaging data; (b) Image format conversion; (c) Brain surface reconstruction and segmentation quality check; (d) Import of CAT12 segmentation results into Brainstorm and generation of three orthogonal views to assess data quality; (e) Generation of a Boundary Element Method (BEM) head model; (f) Construction of a three-layer head surface comprising scalp, skull, and brain tissue; (g) EEG electrode localization; and (h) Integration of electrode position data for final construction of an individualized realistic head model. Supplementary Fig. 5. Flowchart for ESI and brain network analysis. Note: (a) EEG segments were selected and preprocessed; (b) preprocessed data were projected into source space using sLORETA to obtain the spatiotemporal distribution of cortical current density; (c) representative source signals were extracted from different brain regions; (d) a dynamic directed functional network was constructed using DTF; (e) graph theoretical analysis was applied to identify and localize potential EZs; and (f) identified EZs were compared with postoperative resection areas for validation. Supplementary Fig. 6. Schematic diagram of degree centrality metrics. Note: Each circle represents a distinct brain region node within the epilepsy network. Blue arrows denote unidirectional causal connection weights between nodes. Taking Node 1 as an example: DCin refers to the number of incoming connections it receives (2), DCout indicates the number of outgoing connections it sends (1), and the total DC (degree centrality) is the sum of both, totaling 3. Supplementary Fig. 7. Flowchart of SEEG implantation and surgical treatment. Note: (a) SEEG implantation planning was conducted based on noninvasive preoperative evaluation results, followed by electrode implantation surgery; (b) continuous SEEG monitoring was performed to capture both interictal and ictal EEG data; (c) an individualized surgical resection plan was developed according to the SEEG findings; (d) postoperative histopathological diagnosis was obtained; and (e) postoperative cranial CT scans were conducted at regular intervals to assess resection extent and detect potential complications. Supplementary Fig. 8. Multiband PSD analysis of scalp EEG source signals in 15 epilepsy patients. Note: The figure illustrates the power distribution across multiple frequency bands (δ, θ, α, β, low-gamma, and high-gamma) in the EEG signals, highlighting the spectral characteristics of brain activity. The X-axis represents frequency (Hz), and the Y-axis denotes power spectral density (dB/Hz).

## Data Availability

The datasets generated and/or analyzed during the current study are included in the article and its Supplementary Materials. Additional data are available from the corresponding author upon reasonable request.
