# Supplementary figures and images for: A network-based EEG source imaging framework for noninvasive localization of epileptogenic zones in MRI-negative focal drug-resistant epilepsy
Source: BMC Neurol. 2026 Jan 15;26:86. doi: 10.1186/s12883-026-04625-x (PMC12892749; doi:10.1186/s12883-026-04625-x)

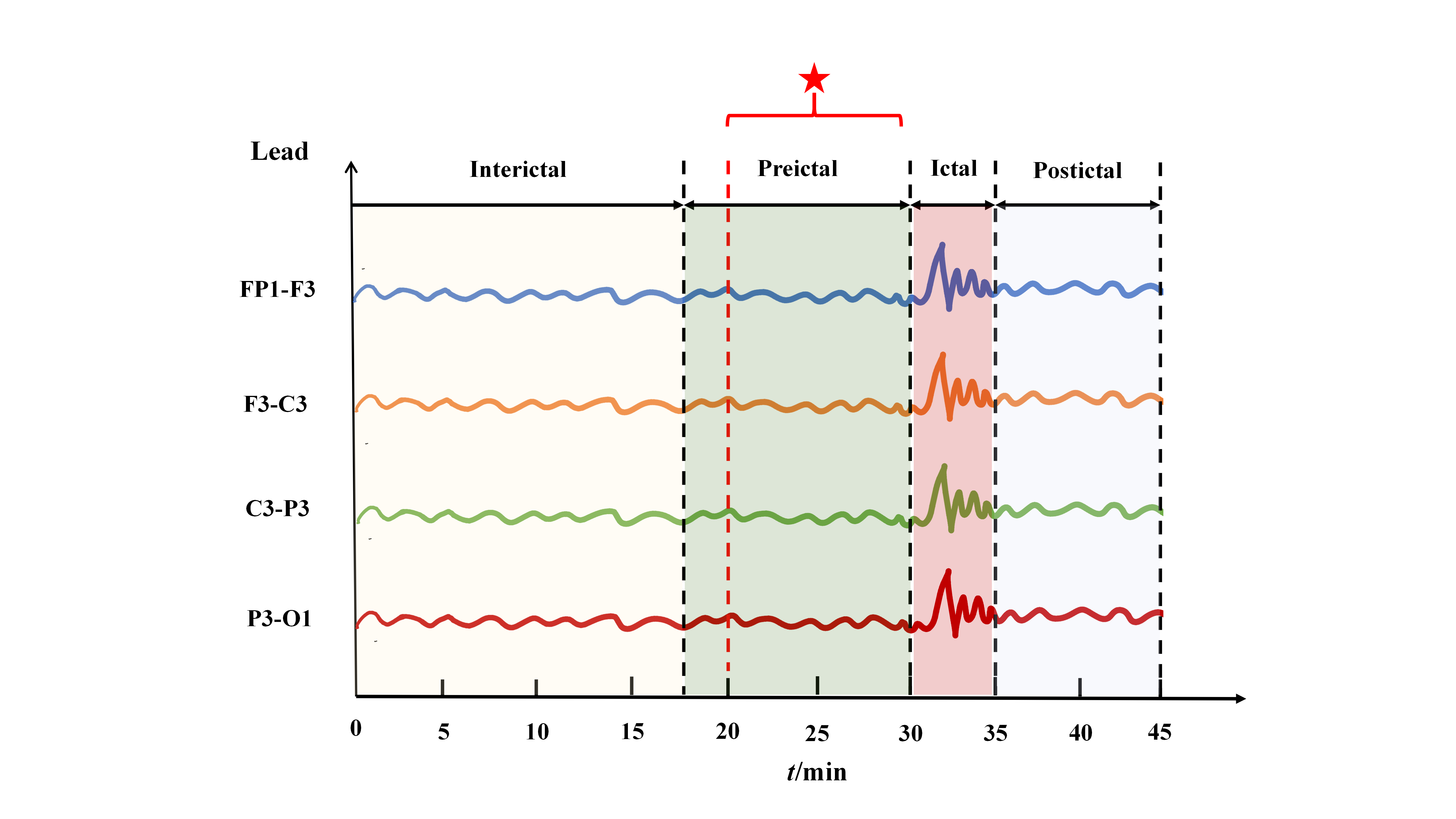

Supplement: Supplementary file 1 — Supplementary Material 1: Supplementary Table 1. Desikan-Killiany Atlas. Supplementary Table 2. Baseline Characteristics of Patients. Supplementary Table 3. Diagnostic Outcome Indicators. Supplementary Fig. 1. Preoperative assessment flowchart. Note: Stage 1a: All patients initially undergo standard non-invasive evaluations. If findings are conclusive, surgical intervention may proceed directly. Stage 1b: For inconclusive or discordant results, additional advanced non-invasive investigations may be performed to refine EZ localization or assess surgical risk. The selection of modalities depends on clinical context and institutional resources. Stage 2: If stage 1b results remain non-localizing or suggest that the EZ is adjacent to eloquent cortex, SEEG combined with cortical stimulation is employed to delineate both the EZ and functional areas. This study involved a retrospective collection of patients’ scalp EEG data and the localization of EZ using ESI, which were subsequently evaluated for concordance with postoperative resection sites and clinical prognosis. Supplementary Fig. 2. Stages of seizure. Note: The horizontal axis represents time, and the vertical axis denotes EEG channel labels. The timeline includes the interictal, preictal, ictal, and postictal phases. In this study, a 10-min segment of EEG data preceding seizure onset—highlighted by red brackets and indicated with a red star—was selected for further analysis. Supplementary Fig. 3. EEG preprocessing. Note: (a) A 10-min EEG segment preceding seizure onset was selected; (b) electrode channel positions were accurately aligned according to the 10–20 system; (c) a band-pass filter (0.5–80 Hz) and a notch filter (48–52 Hz) were applied to remove baseline drift and power line interference, respectively; (d) independent component analysis (ICA) was conducted to isolate noise components; (e) artifacts and identified interference signals were removed; and (f) the cleaned EEG data were segmented into 2-s epochs, [file 12883_2026_4625_MOESM1_ESM.zip › Supplementary Figure 2. Stages of seizure.png]

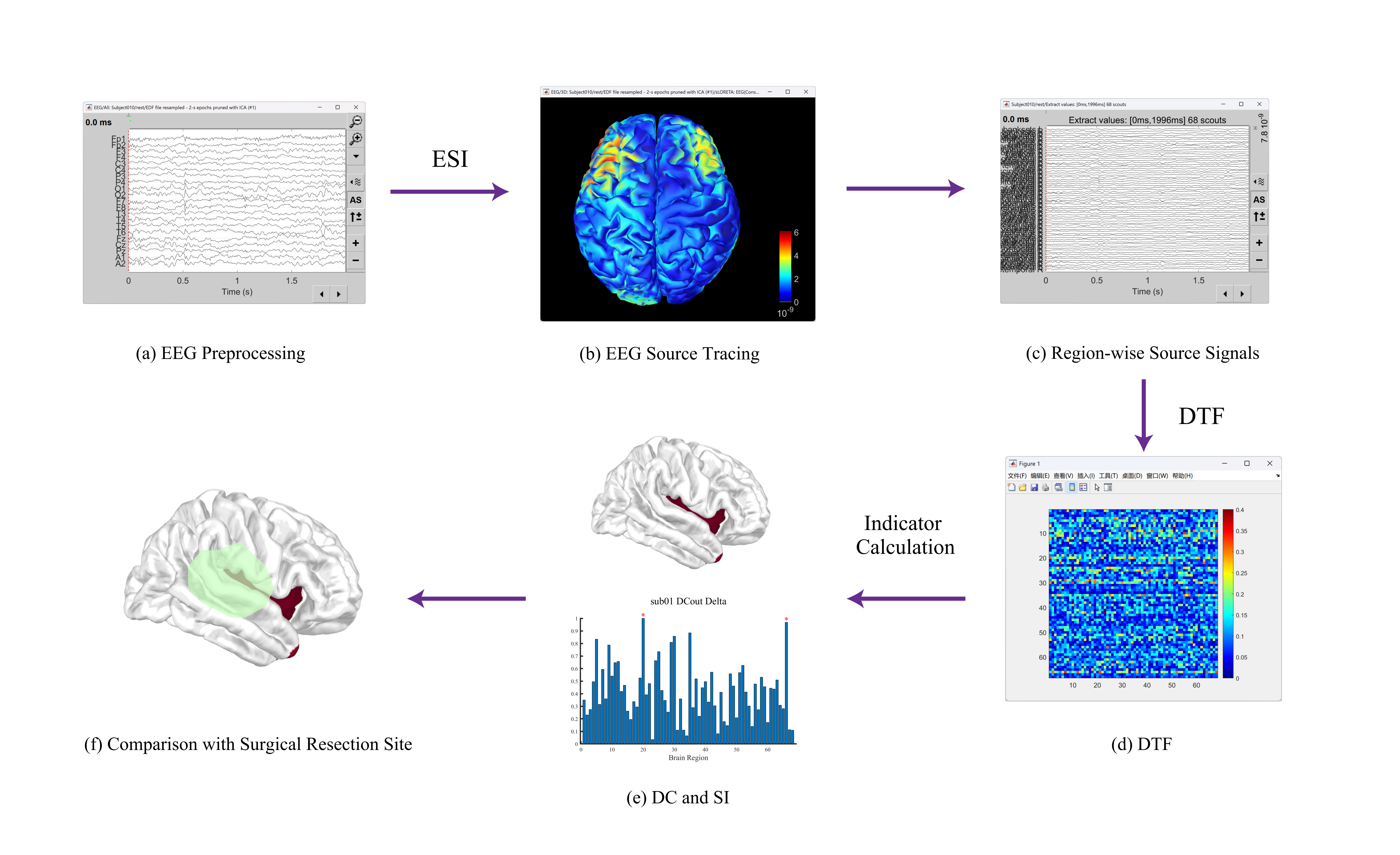

Supplement: Supplementary file 1 — Supplementary Material 1: Supplementary Table 1. Desikan-Killiany Atlas. Supplementary Table 2. Baseline Characteristics of Patients. Supplementary Table 3. Diagnostic Outcome Indicators. Supplementary Fig. 1. Preoperative assessment flowchart. Note: Stage 1a: All patients initially undergo standard non-invasive evaluations. If findings are conclusive, surgical intervention may proceed directly. Stage 1b: For inconclusive or discordant results, additional advanced non-invasive investigations may be performed to refine EZ localization or assess surgical risk. The selection of modalities depends on clinical context and institutional resources. Stage 2: If stage 1b results remain non-localizing or suggest that the EZ is adjacent to eloquent cortex, SEEG combined with cortical stimulation is employed to delineate both the EZ and functional areas. This study involved a retrospective collection of patients’ scalp EEG data and the localization of EZ using ESI, which were subsequently evaluated for concordance with postoperative resection sites and clinical prognosis. Supplementary Fig. 2. Stages of seizure. Note: The horizontal axis represents time, and the vertical axis denotes EEG channel labels. The timeline includes the interictal, preictal, ictal, and postictal phases. In this study, a 10-min segment of EEG data preceding seizure onset—highlighted by red brackets and indicated with a red star—was selected for further analysis. Supplementary Fig. 3. EEG preprocessing. Note: (a) A 10-min EEG segment preceding seizure onset was selected; (b) electrode channel positions were accurately aligned according to the 10–20 system; (c) a band-pass filter (0.5–80 Hz) and a notch filter (48–52 Hz) were applied to remove baseline drift and power line interference, respectively; (d) independent component analysis (ICA) was conducted to isolate noise components; (e) artifacts and identified interference signals were removed; and (f) the cleaned EEG data were segmented into 2-s epochs, [file 12883_2026_4625_MOESM1_ESM.zip › Supplementary Figure 5. Flowchart for ESI and brain network analysisSupplementary Figure 5. Flowchart for ESI and brain network analysis(1).png]

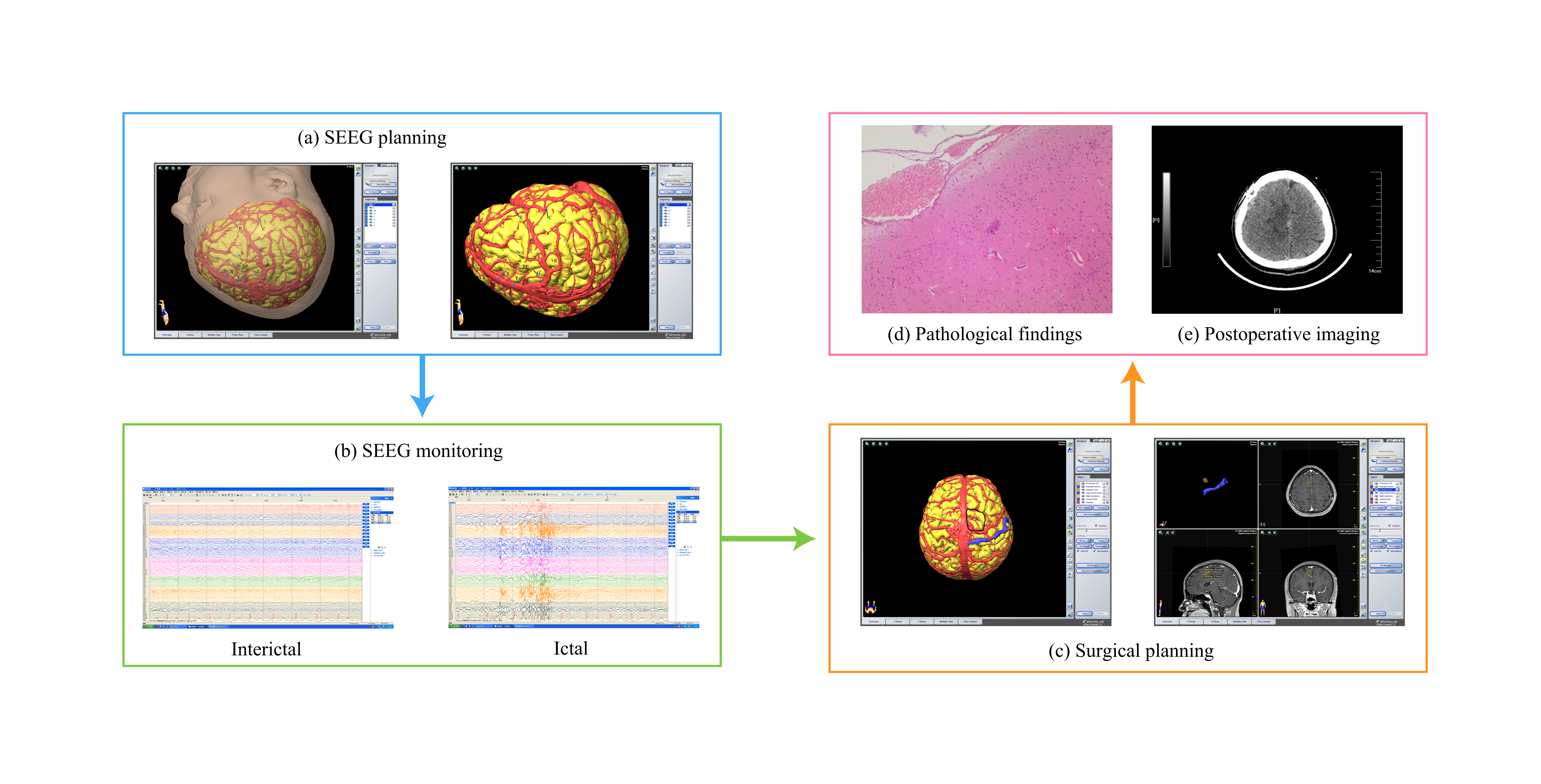

Supplement: Supplementary file 1 — Supplementary Material 1: Supplementary Table 1. Desikan-Killiany Atlas. Supplementary Table 2. Baseline Characteristics of Patients. Supplementary Table 3. Diagnostic Outcome Indicators. Supplementary Fig. 1. Preoperative assessment flowchart. Note: Stage 1a: All patients initially undergo standard non-invasive evaluations. If findings are conclusive, surgical intervention may proceed directly. Stage 1b: For inconclusive or discordant results, additional advanced non-invasive investigations may be performed to refine EZ localization or assess surgical risk. The selection of modalities depends on clinical context and institutional resources. Stage 2: If stage 1b results remain non-localizing or suggest that the EZ is adjacent to eloquent cortex, SEEG combined with cortical stimulation is employed to delineate both the EZ and functional areas. This study involved a retrospective collection of patients’ scalp EEG data and the localization of EZ using ESI, which were subsequently evaluated for concordance with postoperative resection sites and clinical prognosis. Supplementary Fig. 2. Stages of seizure. Note: The horizontal axis represents time, and the vertical axis denotes EEG channel labels. The timeline includes the interictal, preictal, ictal, and postictal phases. In this study, a 10-min segment of EEG data preceding seizure onset—highlighted by red brackets and indicated with a red star—was selected for further analysis. Supplementary Fig. 3. EEG preprocessing. Note: (a) A 10-min EEG segment preceding seizure onset was selected; (b) electrode channel positions were accurately aligned according to the 10–20 system; (c) a band-pass filter (0.5–80 Hz) and a notch filter (48–52 Hz) were applied to remove baseline drift and power line interference, respectively; (d) independent component analysis (ICA) was conducted to isolate noise components; (e) artifacts and identified interference signals were removed; and (f) the cleaned EEG data were segmented into 2-s epochs, [file 12883_2026_4625_MOESM1_ESM.zip › Supplementary Figure 7. Flowchart of SEEG implantation and surgical treatment.png]
